# Xanthogranulomatous pyelonephritis with polycystic kidney disease as a mimic of cystic renal cell carcinoma: a case report

**DOI:** 10.1186/s12894-023-01224-7

**Published:** 2023-04-11

**Authors:** Mei Yi, Yong Liu, Qi Chen

**Affiliations:** Department of Ultrasound, Capital Medical University Affiliated Beijing Shijitan Hospital, No. 10 Tieyi St, Haidian District, Beijing, 100038 China

**Keywords:** Xanthogranulomatous pyelonephritis, Polycystic kidney, Cystic renal cell carcinoma, Case report

## Abstract

**Background:**

Xanthogranulomatous pyelonephritis (XGP) is a rare chronic pyelonephritis that often mimics other renal diseases, when combined with autosomal dominant polycystic kidney disease(ADPKD), preoperative diagnosis is exceedingly difficult. It is important for clinicians to be aware of an XGP with ADPKD since a misdiagnosis can lead to unnecessary surgical intervention.

**Case presentation:**

Here, we report a case of a 66-year-old female with a history of bilateral ADPKD and urinary tract infection admitted to our hospital due to right flank pain, feeble, and low-grade fever. Contrast-enhanced ultrasound revealed a malignant mass of the right kidney suspected to be a cystic renal cell carcinoma with polycystic kidney disease. In addition, contrast-enhanced computed tomography (CT) and fluorine 18 fluorodeoxyglucose PET/CT (^18^F FDG PET/CT) showed similar results. Subsequently, the patient underwent a right radical nephrectomy, but histopathological examination revealed XGP with ADPKD. On the follow-up, the patient's symptoms were relieved.

**Conclusions:**

XGP should be kept in mind during the differential diagnosis of renal masses with ADPKD even in the absence of characteristic clinical symptoms and imaging manifestations.

## Background

Xanthogranulomatous pyelonephritis (XGP) is a severe chronic infectious renal disease, which was first described as lipid-filled macrophages in a granulomatous inflammatory process in 1916 by Schlagenhaufer [1]. XGP can occur at any age, but most often occur in immunocompromised middle-aged women [2, 3]. The exact etiology of XGP remains unclear, and often develops secondary to recurrent urinary tract infections and nephrolithiasis obstructions [4]. This is rare case reporting XGP with ADPKD.

## Case presentation

A 66-year-old female with a history of bilateral ADPKD and urinary tract infection was admitted due to pain in the right flank. The pain in the right flank started approximately 15 days ago, remained persistent, and presented with a low-grade fever and weakness. Although her body temperature was approximately 36.7 °C, she complained of a low basal body temperature throughout the day. There is a family history of ADPKD, but she denied any associated hematuria, dysuria, or weight loss. Physical examination showed no bilateral kidney area bulge or percussed pain. Urinalysis revealed normal red blood cell levels, with white blood cell levels significantly increased to 105.45/μL (normal, 0–28/μL), and the blood tests showed white blood cell levels of 12.8 × 10^9^/L (normal, 3.5 × 10^9^–9.5 × 10^9^/L), red blood cell levels of 3.05 × 10^12^/L (normal, 3.8 × 10^12^–5.1 × 10^12^/L), and elevated C-reactive protein levels (167 mg/L). Renal function tests showed a serum creatinine level of 145 μmol/L (normal, 41-81 μmol/L) and BUN levels, total carbon dioxide, uric acid, and glomerular filtration rate all within normal limits. Urine culture suggested an Escherichia coli (E. coli) infection. Blood culture was not performed. Before admission, the patient underwent conventional ultrasound imaging to determine her symptoms, etiology and assess the right kidney.

**Fig. 1:**
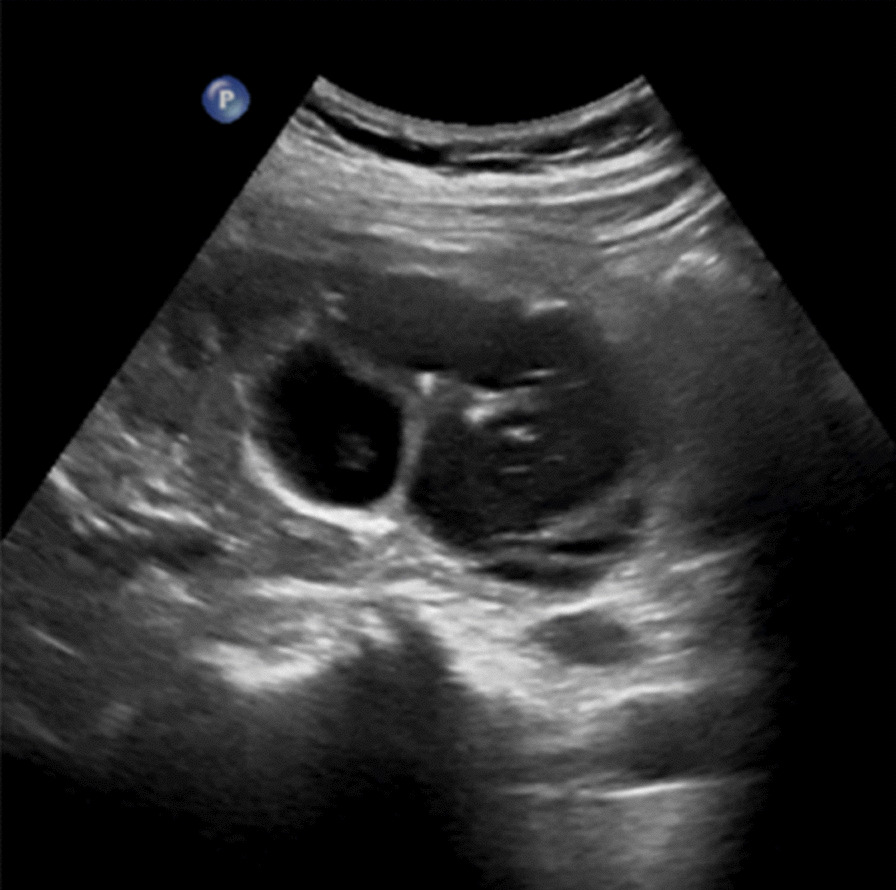
Ultrasonography demonstrating an cystic mass with thickened walls and irregular septa on the middle and the lower pole of the right kidney

**Fig. 2:**
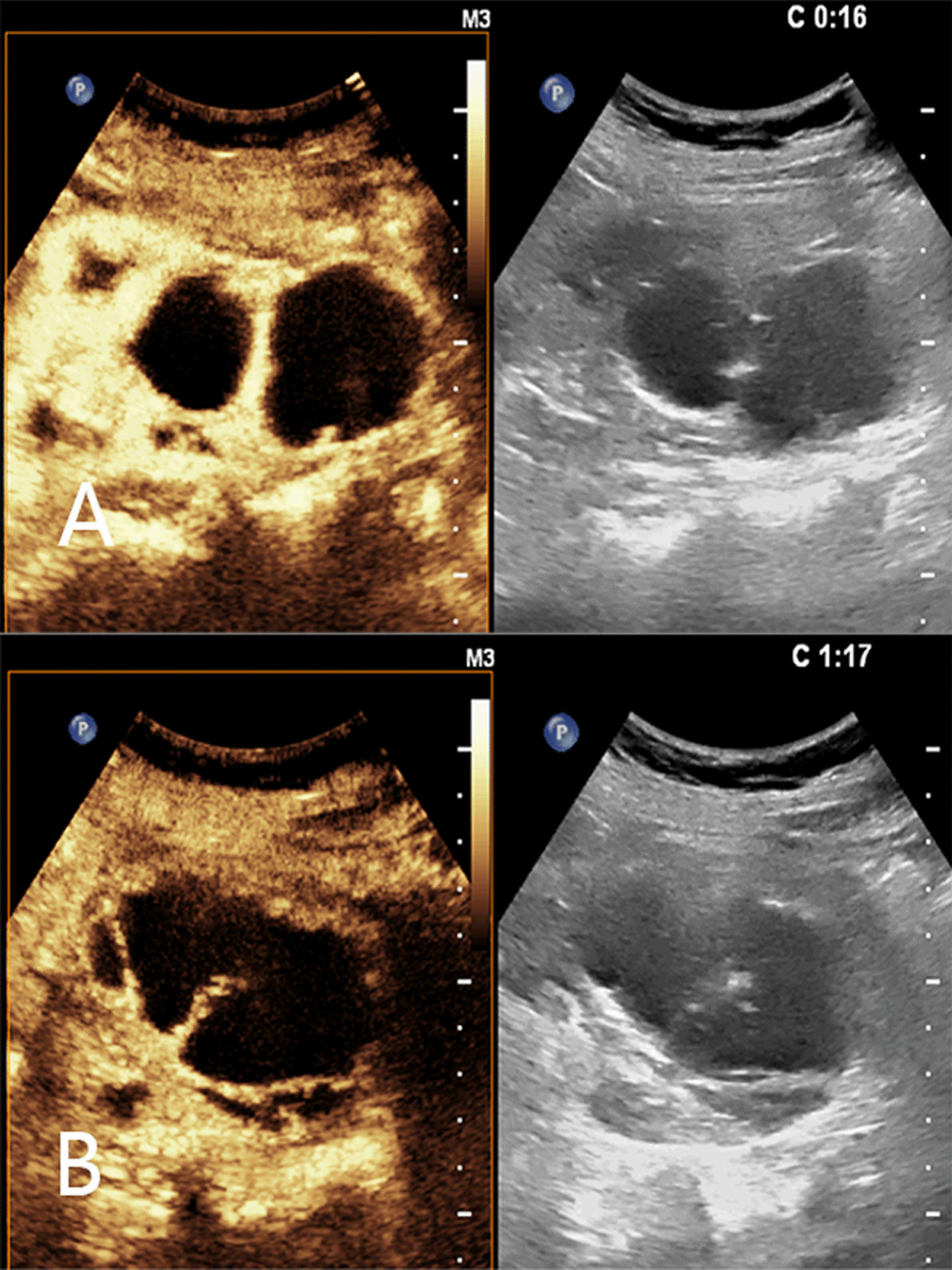
Contrast-enhanced (left) and corresponding gray-scale (right) US images of the right kidney’s cystic mass at **A** 16 s and **B** 1 min 17 s

**Fig. 3:**
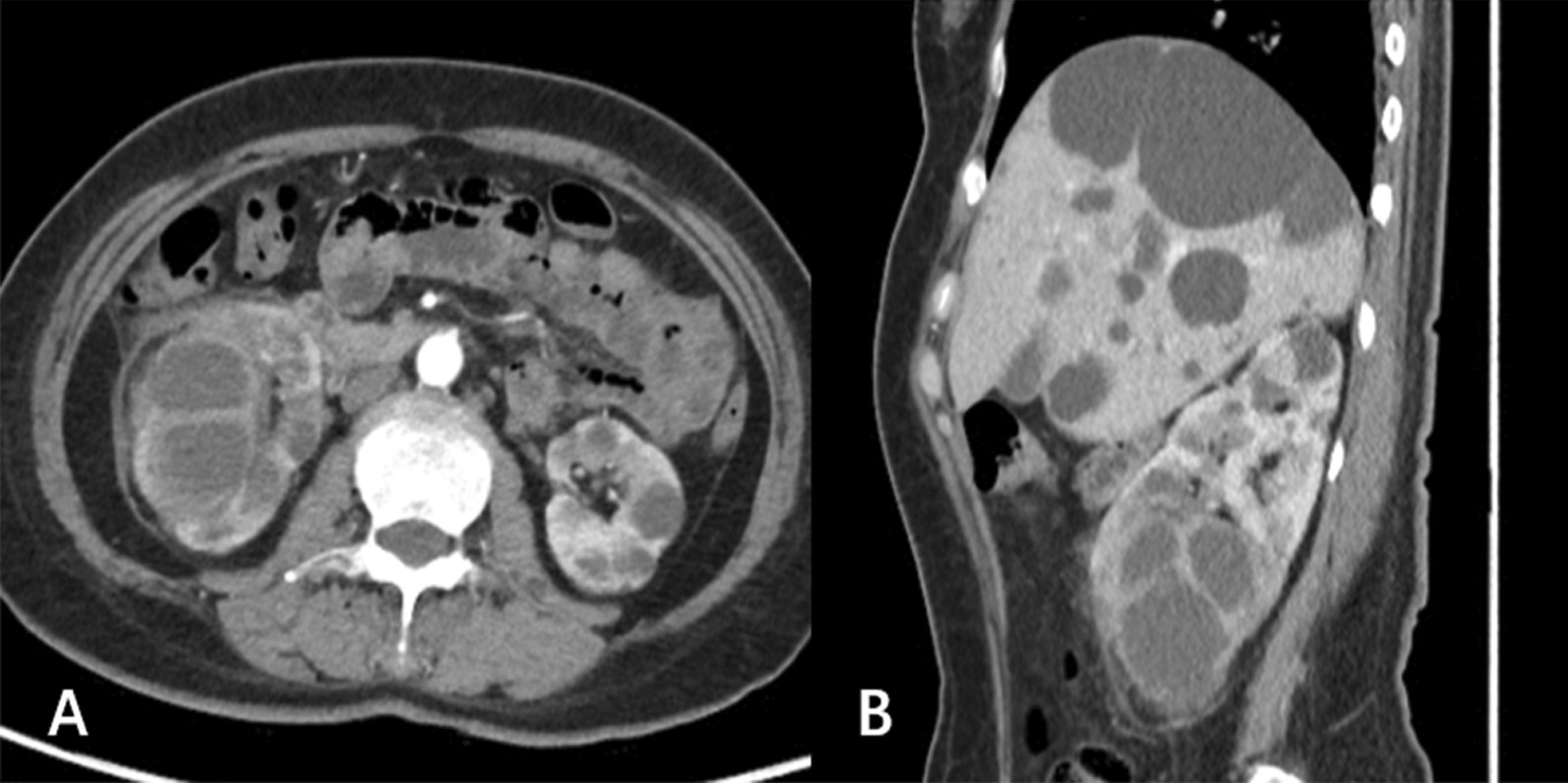
**A** axial and **B** sagittal contrast-enhanced CT images of the middle and the lower abdomen

**Fig. 4:**
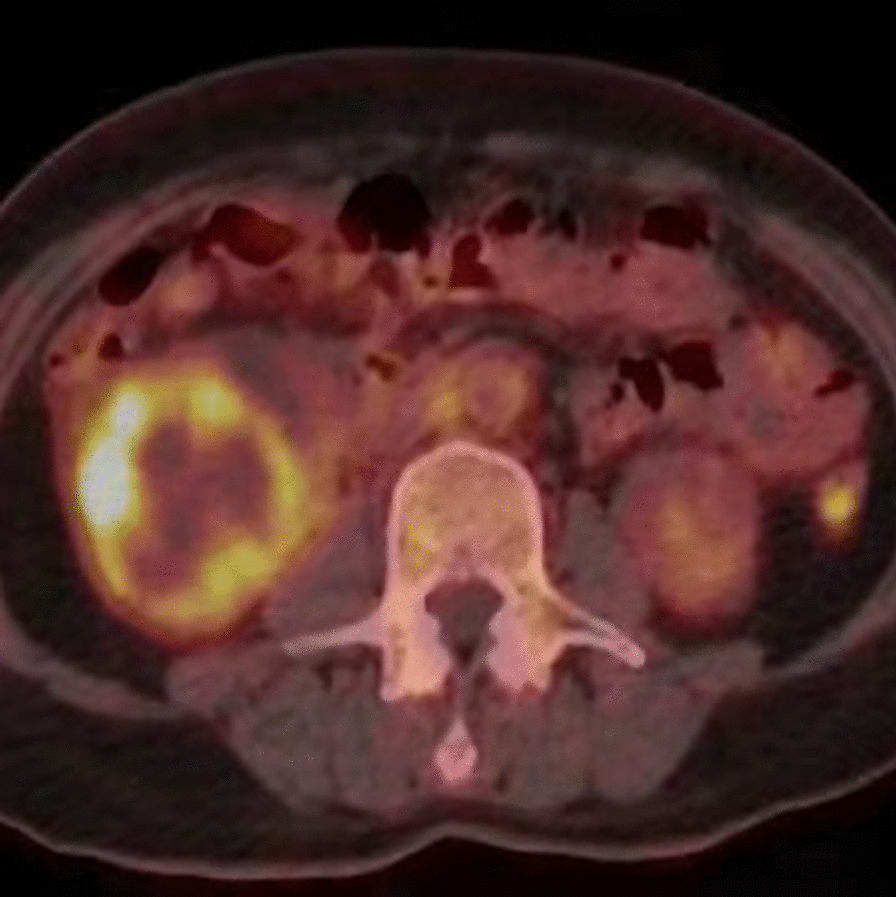
Axial ^18^F FDG PET/CT image of the lower abdomen

Abdominal ultrasound demonstrated a bilateral renal enlargement with multiple diffuse thin-walled cystic masses of various sizes throughout both kidneys, as well as a 7.7 cm × 6.0 cm × 5.7 cm cystic mass thickened walls and irregular septum in the middle and the lower pole of the right kidney (Fig. 1). After CEUS revealed the thickened walls and irregular septations in the cystic mass, there were unequivocal enhancements in the cortical phase, and isoenhancement in cortico-medullary and late phases, the non-enhanced cystic portions of the mass were anechoic (Fig. 2). Thus, the diagnosis of cystic renal cell carcinoma with ADPKD was made. The findings were consistent with contrast-enhanced CT and ^18^F FDG PET/CT. Contrast-enhanced CT images of the lower abdomen showing the enlarged kidneys with multiple cystic low-density shadows of unequal size, there was a separated cystic shadow in the middle and the lower pole of the right kidney, and moderate enhancement of septation at the rims of the cystic shadow (Fig. 3), and axial ^18^F FDG PET/CT image of the lower abdomen showing the FDG-avid the thickened walls in the cystic mass with the FDG-avid post-peritoneum lymph nodes (Fig. 4).

Following an informed discussion, the patient proceeded with a laparoscopic-assisted right radical nephrectomy under general anesthesia to confirm the diagnosis.

**Fig. 5:**
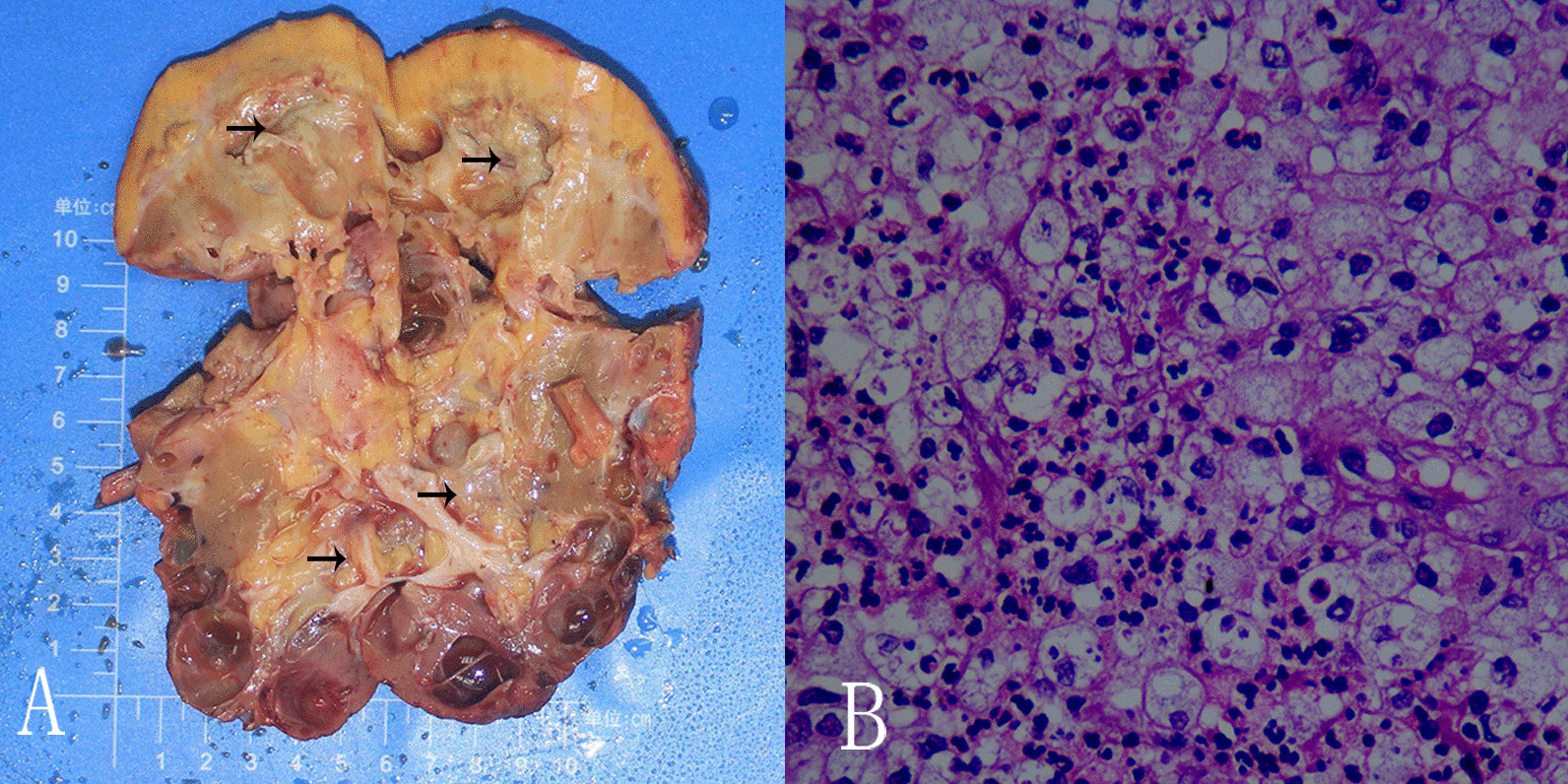
**A** The longitudinal section of the right kidney having XGP with polycystic kidney disease. **B** A granulomatous inflammation with numerous foamy histiocytes, necrotic tissues, as well as neutrophils, lymphocytes and plasma cells infiltrates (Hematoxylin–eosin stain; original magnification, × 400)

Grossly, the right kidney was enlarged in which a large number of cysts and abscesses have replaced most of the normal kidney tissue (Fig. 5A). Histopathological examination of the resected specimen revealed granulomatous inflammation with numerous foamy histiocytes, necrotic tissue, neutrophils, lymphocytes, and plasma cell infiltrates (Fig. 5B). These findings were in accordance with XGP complicated by ADPKD.

No intraoperative or postoperative complications were observed. Antibiotic treatment with intravenous etimicin sulfate was initiated for 6 days after surgery(dose 100 mg intravenously). At six months follow-up, the disease resolved well without any right flank pain, weakness, or low-grade fever, and the BUN and creatinine levels stabilized.

## Discussion and conclusions

XGP is a rare and distinct form of chronic pyelonephritis that usually occurs in the background of a urinary tract obstruction caused by renal calculi and recurrent chronic infections [5]. Its most significant histological feature is the existence of lipid-laden foamy macrophages [6]. XGP can occur at any age, but has the highest incidence in immunocompromised middle-aged women. XPG’s pathology usually presents unilaterally, but occasionally bilaterally in the kidneys. The latter prognosis is worse than the former and is likely due to bilateral renal function impairment [7]. There are usually no characteristic symptoms of XGP.

In our case, there was a history of flank pain, weakness, low-grade fever, urinary tract infections, absence of dysuria, weight loss, nephrolithiasis, renal angle tenderness, and a palpable lump. However, renal complications of ADPKD can also include nephrolithiasis, infection, and pain [8]. Therefore, the preoperative diagnosis of XGP with ADPKD is challenging. This is an extremely rare case reporting XGP with ADPKD.

Imaging helped with the preoperative diagnosis of XGP. According to the imaging, XGP can be divided into two anatomic forms: the diffuse form and the focal form [9]. The diffuse form is the most common. The typical imaging signature is the “bear paw sign,”in which multiple rounded foci filled with lipid-laden macrophages dilated the renal pelvis and calices [10]. Our case did not observe the bear paw sign. Depending on the inflammation’s progression, XGP starts in the renal parenchyma and the perirenal adipose space and subsequently spreads to adjacent structures or the retroperitoneum, culminating in the formation of renoduodenal fistula, pancreatic adhesions or nephrocutaneous fistula [11–13]. Preoperative imaging should thoroughly evaluate the relationship of kidneys to the surrounding tissue and organs in order to prevent serious complications [14].

XPG has a close resemblance, clinical symptoms, and imaging findings to that of a renal tumor in the focal form, also known as the pseudotumor form [15]. Ultrasonic manifestations of renal cell carcinoma are round or oval solid space-occupying lesions, with complex internal echoes, but generally smooth edges and clear boundaries, and some of them can see low vocal cords. The focal form of XGP is a chronic inflammation, and the boundary of the lesions is vague. It can be preliminarily identified in combination with signs such as renal calculi and perirenal involvement.

In this case, the initial presumptive diagnosis was a cystic renal cell carcinoma with polycystic kidney disease. These results raised concerns about renal malignancies, including unequivocal enhanced masses in the right kidney imaged using CEUS and contrast-enhanced CT,the FDG-avid revealed the thickened walls in the cystic mass with the FDG-avid post-peritoneum lymph nodes imaged via ^18^F-FDG PET/CT. Meanwhile, this patient had a history of bilateral ADPKD, which further increased the preoperative diagnostic difficulty. However, if the focal XPG combined with ADPKD is misdiagnosed as a malignant renal tumor, it will lead to an unnecessary surgical resection. For focal XPG combined with ADPKD, especially patients with severe renal insufficiency or a solitary kidney we advocate an ultrasound-guided puncture biopsy of the focal mass in order to make a clear diagnosis before operation[16–18].

Beyond that, XGP might also mimic other renal tumors, renal tuberculosis, and renal abscesses. Nevertheless, lipid-laden foamy macrophages are typical and necessary for the diagnosis of XGP, and can be histopathologically differentiated from other renal diseases.

The recommended treatment of XGP includes surgery and antibiotic therapy. Surgery includes both an open and laparoscopic nephrectomy. Asali et al. [19] concluded that laparoscopic nephrectomy should be considered an initial approach for XGP. The indications for laparoscopic nephrectomy should be extended to these patients. Antibiotic therapy can serve as an alternative therapy for small and focal lesions.

In conclusion, XGP with polycystic kidney disease is extremely rare. The purpose of this case study is to emphasize that XPG should be taken into consideration in the differential diagnosis of renal masses with polycystic kidney disease, even in the absence of characteristic clinical symptoms. Careful preoperative examination plays an important role in choosing the treatment direction and operation mode.

## Data Availability

All data generated or analysed during this study are included in this published article.

## Abbreviations

XGP: Xanthogranulomatous pyelonephritis
ADPKD: Autosomal dominant polycystic kidney disease
CT: Computed tomography
^18^F FDG PET/CT: Fluorine 18 fluorodeoxyglucose PET/CT
CEUS: Contrast-enhanced ultrasound
